# Genome-Wide Association Analysis for Candidate Genes Contributing to Kernel-Related Traits in Maize

**DOI:** 10.3389/fpls.2022.872292

**Published:** 2022-05-24

**Authors:** Zhibo Qu, Ying Wu, Die Hu, Ting Li, Hangyu Liang, Fan Ye, Jiquan Xue, Shutu Xu

**Affiliations:** ^1^Key Laboratory of Biology and Genetic Improvement of Maize in Arid Area of Northwest Region, Ministry of Agriculture and Rural Affairs, College of Agronomy, Northwest A&F University, Yangling, China; ^2^Maize Engineering Technology Research Centre, Yangling, China

**Keywords:** genome-wide association study, expression profile, kernel-related traits, maize, differential expression

## Abstract

Maize grain size is the main factor determining grain yield. Dissecting the genetic basis of maize grain size may help reveal the regulatory mechanism of maize seed development and yield formation. In this study, two associated populations were used for genome-wide association analysis of kernel length, kernel width, kernel thickness, and hundred-kernel weight from multiple locations in AM122 and AM180, respectively. Then, genome-wide association mapping was performed based on the maize 6H90K SNP chip. A total of 139 loci were identified as associated with the four traits with *p* < 1 × 10^−4^ using two models (FarmCPU and MLM). The transcriptome data showed that 15 of them were expressed differentially in two maize-inbred lines KB182 (small kernel) and KB020 (big kernel) during kernel development. These candidate genes were enriched in regulating peroxidase activity, oxidoreductase, and leaf senescence. The molecular function was major in binding and catalytic activity. This study provided important reference information for exploring maize kernel development mechanisms and applying molecular markers in high-yield breeding.

## Introduction

As one of the most important crops, maize (*Zea mays* L.) is cultivated worldwide and plays an important role in food security and social development because of its increasing demand in food, animal feed, biofuel utilization, and industry (Godfray et al., 2010). Considering the continuous population growth, environmental deterioration, and decrease in arable land, maize yield urgently needs to be increased to ensure food security. Maize grain yield is a complex trait that can be divided into smaller components, including kernel-related traits. Due to the stable heritability (Messmer et al., 2009; Raihan et al., 2016), the improvement in kernel-related traits during new variety breeding can be used for increasing grain yield with the help of biotechnology. Therefore, the genetic basis for breeding high-yield hybrids needs to be clarified.

For kernel-related traits, many genes were characterized and cloned using comparative genomics and mutants, such as *ZmGW2* (Li et al., 2010b), *ZmGS3* (Li et al., 2010a), *defective kerne*l (dek) mutants (Demko et al., 2014; Qi et al., 2017, 2019; Wang et al., 2017; Dai et al., 2018; Li et al., 2018; He et al., 2019), *MN2* (Guan et al., 2020), and *ZmCEP1* (Xu et al., 2021). Many studies identified QTLs or associated SNPs to dissect the genetic mechanism of kernel-related traits. For instance, 26 stable QTLs and 6 stable SNPs were detected across multiple environments for eight ear and kernel morphological traits in maize using combined linkage and association mapping (Zhang et al., 2017). A total of 729 QTLs related to kernel size and kernel weight were identified from 10 recombinant inbred lines (RILs) (Liu et al., 2017). Seventy-three candidate genes regulating seed development were identified by combined linkage and association mapping (Liu M. et al., 2020). A kernel weight QTL (*qKW9*) was identified and fine mapped in Zheng 58/SK RIL populations (Raihan et al., 2016). Then, *qKW9* was cloned, which was found to affect kernel weight through photosynthesis and grain filling (Huang et al., 2020). Meanwhile, the embryo size and related traits, which contributed to kernel size, kernel weight, and kernel nutrition, were also evaluated in nested association mapping populations, and 222 QTLs were identified using genome-wide association analysis (GWAS) (Li W. et al., 2021). Zhou et al. performed novel kernel size analysis for developing maize kernels by integrated GWAS and protein QTL approach (Zhou et al., 2021).

Furthermore, the plasticity and heterosis of kernel-related traits were also analyzed. Li et al. reported that the mean phenotype or plasticity of the hundred-kernel weight and volume was commonly regulated to a high degree, and the plasticity of kernel size and weight might be indirectly selected during maize breeding (Liu Y. et al., 2020). Liu et al. evaluated the mid-parent heterosis for six kernel-related traits and concluded that dominance was more important than other genetic effects for heterosis for kernel-related traits (Liu M. et al., 2020). The genetic architecture of maize embryo size and its related traits appeared to be dominated by multiple small-effect loci with little epistasis. The results showed that the genetic control underlying embryo size appeared to be distinct from that underlying kernel size (Li X. et al., 2021).

However, still more work needs to be done to understand the genetic regulation of kernel-related traits due to the complex genetic components and limited population. In this study, we conducted GWAS for four kernel-related traits in a new association mapping population. The collected inbred lines were still used widely for variety breeding in the northwest of China. Our objectives were to (1) assess the natural variation in the four kernel-related traits in the new natural population, (2) identify novel putative genes for kernel-related traits in this new population, and (3) provide a potential reference value for northwest maize breeding.

## Materials and Methods

### Plant Materials and Field Experiment

In this study, a new association panel was constructed using 205 excellent inbred lines, which were collected from breeders and belonged to the maize in the northwest of China. Most of them were used as parents of authorized hybrids in China. Of these, 122 inbred lines (AM122) and 180 inbred lines (AM180) were collected in 2019 and 2020, respectively. Subsequently, AM122 was planted in Yulin (19YL), Ningxia (19NX), and Urumqi (19UR) in 2019, and AM180 was planted in Ningxia (20NX), Urumqi (20UR), Taiyuan (20TY), and Zhangye (20ZY) in 2020. For each material, a two-row plot was designed, 3 m in length and with a spacing of 0.2 m between plants and 0.6 m of row interval. The field was managed according to the standard agronomic practices for maize.

### Phenotype Collection and Analysis

The kernel-related traits, including kernel length (KL), kernel width (KW), kernel thick (KT), and hundred-kernel weight (HKW), were measured after harvest and air-dried. The descriptive statistical analysis between the traits was carried out using IBM SPSS statistics 23 software and then visualized using Origin 2021 (http://www.OriginLab.com). The formula *H*^2^ = $\sigma_{\text{g}}^{2}$/($\sigma_{\text{g}}^{2}$ +$\sigma_{\text{e}}^{2}$/*l*) and *H*^2^ = $\sigma_{\text{g}}^{2}$/($\sigma_{\text{g}}^{2}$ + $\sigma_{\text{gy}}^{2}$/*l* +$\sigma_{\text{e}}^{2}$/*lr*) were used to estimate heritability, where $\sigma_{\text{g}}^{2}$ is genetic variance, $\sigma_{\text{gy}}^{2}$ is the variance of genotype and environment interaction, $\sigma_{\text{e}}^{2}$ is the error variance, *l* is the number of locales, and *r* is the number of repeats (Knapp et al., 1985). We used the R package (http://www.r-project.org/) *lme4* to estimate a predicted value for each trait by best linear unbiased prediction (BLUP) to eliminate the influence of environmental factors on phenotypes.

### Genotyping and Genome-Wide Association Mapping

Fresh young leaves of the AM205 in the five-leaf stage were sampled and grounded to powder in liquid nitrogen. Then, genome DNA was extracted using the modified CTAB method (Murray and Thompson, 1980), and the genotype was detected using the SNP 6H90K chip platform (Beidahuang Kenfeng Seed Industry Co., Ltd., Harbin, China). After deleting the low-quality or rare SNPs with missing data > 20% and minor allele frequency (MAF) < 5% by PLINK tool (Purcell et al., 2007), 42,049 SNPs were left in AM122 and 42,526 SNPs in AM180. The genetic background of 205 inbred lines was analyzed by principal component analysis (PCA) using R-packages GCTA (http://www.r-project.org/). GWAS was carried out with an MLM model in Tassle 5.0 (https://nl.aliexpress.com/) and a Farm CPU model by GAPIT in the R package (https://zzlab.net/GAPIT/) to balance false positive and false negatives.

### Candidate Gene Function Mining

The expression level of genes nearest to the associated SNPs was analyzed using two data sets to explore the candidate genes. The first set comprised the B73 (the reference genome in maize) expression data downloaded from the public database (https://maizegdb.org). The other data set were from RNA sequences data of kernels in two inbred lines, including KB182 (small size with low kernel weight) and KB020 (big size with high kernel weight) in six developmental stages, including 21 days after pollination (21DAP), 28DAP, 35DAP, 42DAP, 49DAP, and 56DAP. The genes were regarded as differentially expressed using the cutoff criteria of an FDR-adjusted *P* ≤ 0.05 and a fold change (FPKM) ≥ 2 or ≤ 0.5. Then, the candidate genes were clustered and visualized using R-packet *pheatmap* (http://www.r-project.org/).

The high-expression candidate gene protein interaction network was analyzed using STRING v11.0 (https://string-preview.org/) and visualized using Cytoscape v3.8.0 (http://www.cytoscape.org/download.php). Finally, the enrichment analysis of Gene Ontology (GO) terms was performed using the online tool AgriGO (v2.0) (http://systemsbiology.cau.edu.cn/agriGOv2/) (Du et al., 2010).

## Results

### Phenotypic Variation in Kernel-Related Traits in the Association Panel

**Figure 1:**
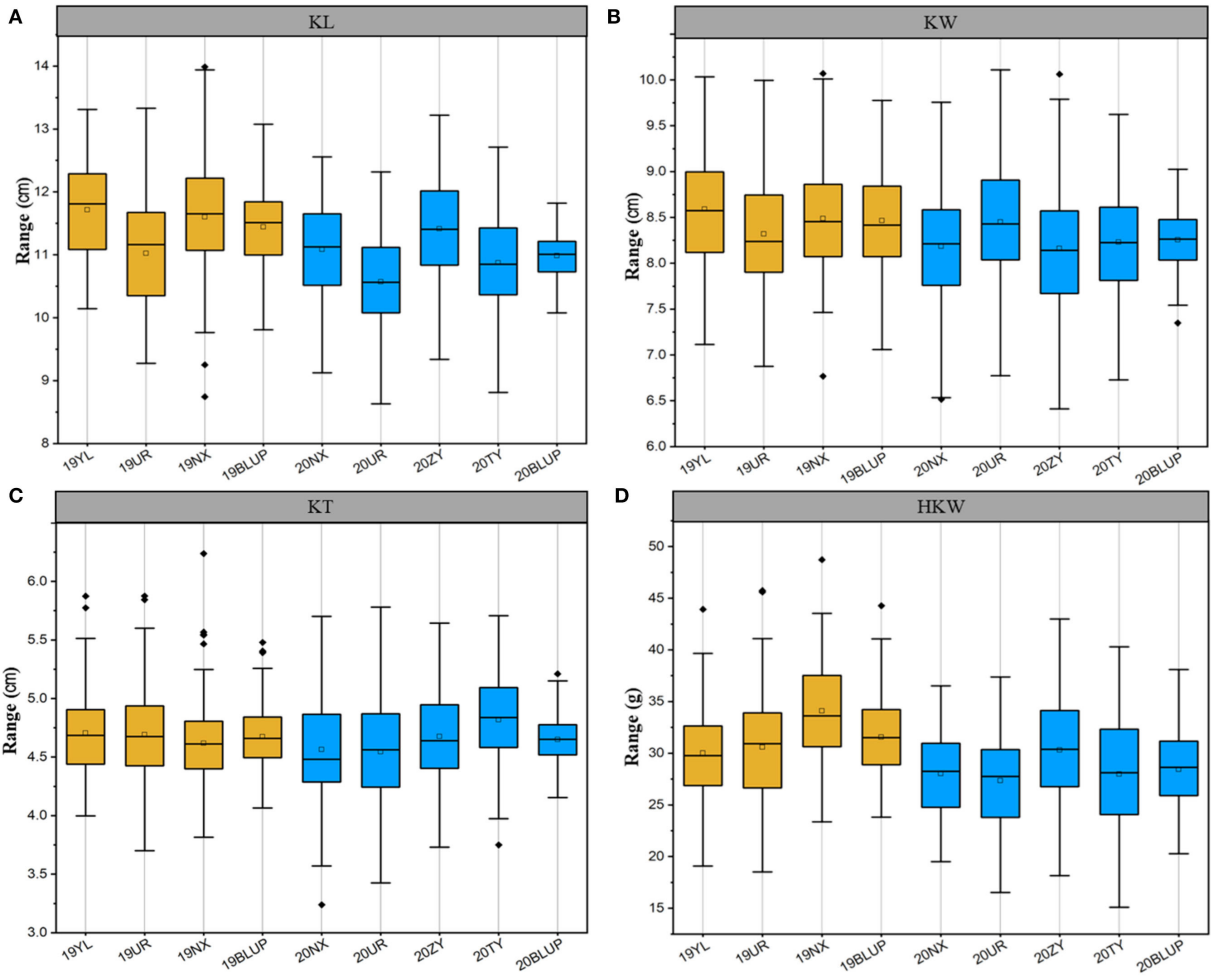
Phenotypic distribution of the kernel-related trait in different environments. **(A)** KL, Kernel length; **(B)** KW, kernel width; **(C)** KT, kernel thickness; **(D)** HKW, hundred-kernel weight. YL, Yulin; UR, Urumqi; NX, Ningxia; ZY, Zhangye; TY, Taiyuan; BLUP, Best Linear Unbiased Prediction. Dark yellow represents the location in 2019, and blue represents the location in 2020.

AM122 and AM180 were planted in multiple locations in 2019 and 2020, respectively, to evaluate the phenotypic variation in kernel-related traits of inbred lines from the northwest in China. Four related traits, including KL, KW, KT, and HKW, were collected. They all presented a normal distribution and were basically consistent in the 2 years (Figure 1, Supplementary Table S1). The coefficient of variation (CV) for traits was 6.66% to 17.09% in AM122 and from 6.56 to 18.68 in AM180. In both association panels, the highest CV was present in HKW, which was affected by KL, KT, and KW. The broad heritability of the four traits ranged from 84.93 to 90.96% in AM122. It was higher than that in AM180, ranging from 63.90 to 86.38% (Supplementary Table S1).

The correlation coefficient for one trait in the same association panel significantly positively correlated between different locations, which was greater in AM122 than in AM180. It was consistent with the performance of heritability between AM122 and AM180. In addition, the correlation between locations for the same trait was higher than that between different traits in the same location (Supplementary Table S1). Due to the most of the materials were different in the two-association panel, hence, the BLUP value of each line was estimated across environments in AM122 and AM180 to reduce the environmental noise. They basically followed normal distribution, with a CV range of 3.11–12.54% for the four traits in two association panels (Supplementary Table S1)

### GWAS of Kernel-Related Traits

**Figure 2:**
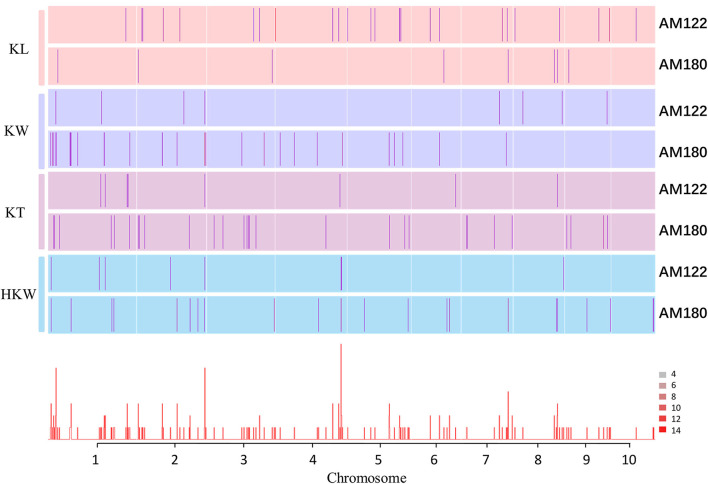
Distribution of associated SNPs of four kernel-related traits using two models (FarmCPU and MLM). The vertical line indicates the position of the locus associated with the grain size trait on the chromosome; The scale bar indicates the significance level of association sites. The height of the histogram represents the frequency of the site. AM122 represents SNPs significantly related to all locations in 2019 (19YL, 19UR, 19NX, and 19BLUP); AM180 represents SNPs significantly related to all locations in 2020 (20NX, 20UR, 20ZY, 20TY, and 20BLUP). KL, Kernel length; KW, kernel width; KT, kernel thickness; HKW, hundred-kernel weight.

Genome-wide association mapping was carried out in AM122 and AM180 to explore the genetic basis of the four kernel traits. Through principal component analysis and group structure analysis, it can be found that AM205 is divided into eight subgroups, and the contribution rates of PC1, PC2, and PC3 are 13.59, 5.91, and 4.02%, respectively (Supplementary Table S2). Two methods, including FarmCPU by GAPIT in the R package and MLM in TASSEL, were used to identify the associated loci (see details in the Materials and Methods section) based on the maize 6H90K SNP chip. After filtering low-quality SNPs with missing rates >0.2 and minor allele frequency <0.05, 42,049 and 42,526 high-quality SNPs were left in AM122 and AM180, respectively. When setting the threshold *P*-value for associated SNPs as 1/n (n is the number of SNP markers) or 0.05/n, it is too strict to discover the significant sites due to the particularity of this population, which were with existing breeding germplasm, according to a previous study (Wang et al., 2012; Zhao et al., 2018). We set the threshold to 1 × 10^−4^. Based on two models, twenty-six, eight, nine, and nine SNPs significantly related to KL, KW, KT, and HKW were detected in AM122 (Figure 2, Supplementary Table S2). Of which, four, two, three, and four for the four traits were identified more than two times, including multiple environments, two models and two traits. In AM180, 10, 31, 29, and 25 SNPs were identified for the four kernel traits (Supplementary Figure S4, Supplementary Table S2), of which one, three, two, and six associated SNPs were overlapped under two models or environments or two traits, respectively (Supplementary Table S2).

Together, 139 unique loci were found to be associated with the four kernel traits with 7.23% average phenotypic variation explained (Supplementary Table S2). Furthermore, 30 of them could explain more than 10% phenotypic variation, which were usually considered to work as major effect genes. Furthermore, 20 of them were detected two times or more. For instance, the SNP Affx-158854316 markers were associated with KW in AM122 (BLUP and 19YL) and AM180 (20ZY), and HKW in AM122 (BLUP and 19NX) and KT in AM122 (19YL). Affx-291440710 markers were detected among significant markers related to HKW (BLUP and 20ZY) and KL (20ZY) in AM180. Affx-291384181 markers were related to KL at NX in AM122 using FarmCPU and MLM. Obviously, four SNPs, including Affx-291429931 (chr4:224307693), Affx-88987667 (chr4:224366960), Affx-291410507 (chr4:224414845), and Affx-159049531 (chr4:225885794), were detected with HKW at least two times. The first SNPs were identified by MLM, the middle two by FarmCPU, and the last SNP by both methods. Although the linkage disequilibrium (LD) decay of this panel was not estimated at the genome-wide level, there were many articles reported the LD decay distance that were usually 10–200 kb in maize. Therefore, we inferred that there would be more than one gene in this 1.5-MB region (chr4:224307693-225885794) (Supplementary Table S2). Certainly, it needs more proof to be verified in the further research.

### Candidate Genes for the Four Kernel-Related Traits in Maize

**Figure 3:**
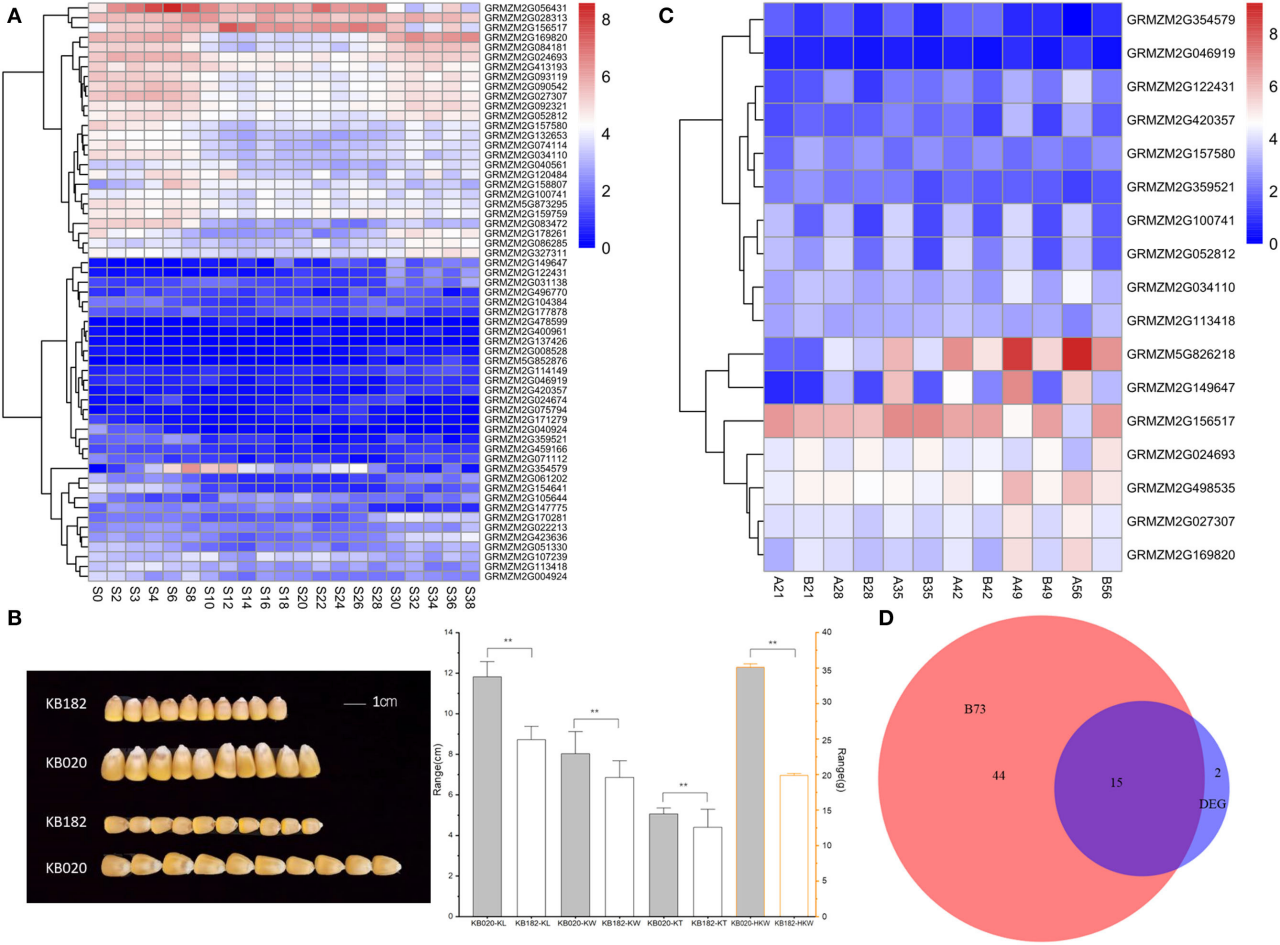
Expression profile of the candidate genes and phenotype of KB182 and KB020. **(A)** Dynamic expression patterns of candidate genes related to four traits in B73 during kernel development. S denotes days after pollination for the seed. **(B)** Kernel images of maize inbred lines KB182 and KB020. Ten seeds each was taken and bar graph of the distribution of three kernel-related traits in KB182 and KB020. The Vernier caliper was used to measure 20 kernels with uniform size. KL, kernel length; KW, kernel width; KT, kernel thickness. **Indicates significance at the level of *P* < 0.01. The data range of KL, KW, and KT is the scale of the left axis. The data range of HKW is the scale of the right axis. **(C)** Dynamic map of kernel development of candidate genes determined using significant SNPs with different genotypes in maize inbred lines KB182 (small kernel) and KB020 (big kernel). In the ordinate, A represents KB182, B represents KB020, and the number represents the days after pollination. **(D)** Common identified genes in B73 and the comparison of KB182 and KB020. The scale bars indicate the relative expression levels of genes. B73 represents the number of genes in **(A)**/ DEG represents the number of genes in **(C)**.

The genes nearest to the 139 significant loci were considered as the potential candidate genes, according to the physical position in the B73 genome (ZmB73_RefGen_v3; http://www.maizesequence.org/), to explore the potential candidate genes for the four kernel-related traits. Finally, 134 candidate genes were reserved because five SNP pairs (Affx-291428872 and Affx-291440710, Affx-291398122 and Affx-291444733, Affx-291398371 and Affx-291381235, Affx-291380474 and Affx-291429145, and Affx-291423841 and Affx-158835632) were located in the same gene (Supplementary Table S3). Combined with the transcriptome data of kernel development in the B73 reference, only 59 genes of these 134 candidate genes were expressed with FPKM ≥ 1 during kernel development from 0DAP to 38DAP in a previous study (Chen et al., 2014; Figure 3A).

Two inbred lines (KB182 and KB020), which had a significant difference in kernel-related traits were (Figure 3C), subjected to RNA sequencing using kernels from 21DAP to 56DAP with a 7-day interval to further clarify the expression profile. For KL, KW, KT, and HKW, the phenotype in KB182 was smaller with lower HKW than that in KB020 (Figure 3B). Among the 134 candidate genes identified using GWAS, 17 were expressed differentially between the two inbred lines during kernel development (Figure 3D). Moreover, 15 genes were among the 59 expressed genes in B73, including *GRMZM2G149647*-annotated heat shock protein26 (*HSP26*) and *GRMZM2G169820*-annotated auxin response factor 21 (*ARF21*, Figure 3D, Table 1). We focused on these for further analysis.

**Table 1:** Candidate genes related to KL, KW, KT and HKW.

**SNP**	**Candidate gene_V3**	**Candidate gene_V4**	**Position**	**Chr**.	**Trait**	**Annotation**	**Module**
Affx-291390256	GRMZM2G149647	Zm00001d028408	33464034	1	KL	heat shock protein 26	M1
Affx-291402221	GRMZM2G024693	Zm00001d017026	27377500	5	KL	Ras-related protein Rab-2-B-like	M2
Affx-291397929	GRMZM2G052812	Zm00001d036024	159765889	6	KL	IN2-2 protein;PCO148860;PCO148860a;aip1; aldo/keto reductase AKR1;auxin induced protein homolog1;auxin-induced protein PCNT115;csu190(gfu);csuh190;pco 148860(579);putative oxidoreductase, aldo/keto reductase family protein	M3
Affx-291422408	GRMZM2G156517	Zm00001d037052	224307693	6	KL	putative ubiquitin-conjugating enzyme family	M4
Affx-291437566	GRMZM2G034110	Zm00001d000184	64103454	9	KL	Transcription factor MYBS3	M5
Affx-291440710	GRMZM2G100741	Zm00001d021873	181477160	7	KL/HKW	uncharacterized protein LOC100277857	M6
Affx-291406950	GRMZM2G046919	Zm00001d027611	8974336	1	KW	pentatricopeptide repeat-containing protein At5g08305	M7
Affx-291429145	GRMZM2G157580	Zm00001d028237	178786938	1	KW	uncharacterized protein LOC103632821	M8
Affx-291443459	GRMZM2G169820	Zm00001d031522	115518026	1	KW	auxin response factor 21	M9
Affx-291399559	GRMZM2G027307	Zm00001d004169	229506045	2	KW	palmitoyltransferase ZDHHC20	M10
Affx-291383773	GRMZM2G420357	Zm00001d027998	19400024	1	KT	leucine-rich repeat extensin-like protein 3	M11
Affx-291398282	GRMZM2G122431	Zm00001d041972	145210669	3	KT	cellulose synthase-like protein G2	M12
Affx-291407617	GRMZM2G113418	Zm00001d018192	87482064	5	KT	glutaredoxin 2	M13
Affx-291375641	GRMZM2G359521	Zm00001d005752	190584813	2	HKW	uncharacterized protein	M14
Affx-291381677	GRMZM2G354579	Zm00001d044607	27905121	3	HKW	uncharacterized protein LOC103651651	-

*Module indicate the protein-protein interaction network of the proteins encoded by candidate genes*.

*KL, kernel length; KW, kernel width; KT, kernel thickness; HKW, hundred-kernel weight; Chr., chromosome*.

### Regulatory Network of Candidate Genes

**Figure 4:**
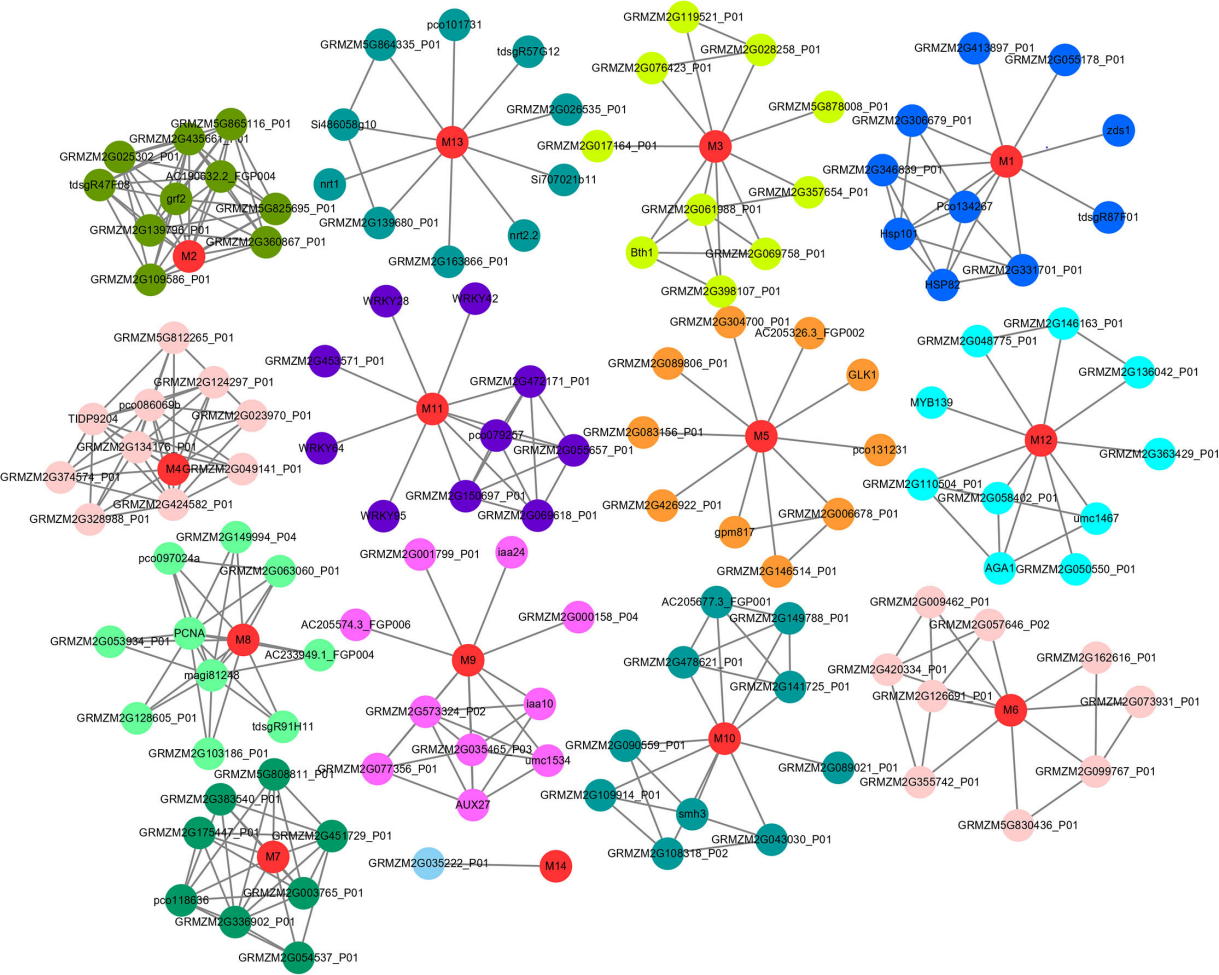
Protein-protein interaction networks of 14 candidate genes. The nodes indicate proteins, and the lines indicate the interaction between proteins. Red color indicates candidate genes encoded proteins, and the interactive proteins in different networks are distinguished by different colors. M1-M14 are the candidate genes encoded proteins.

**Figure 5:**
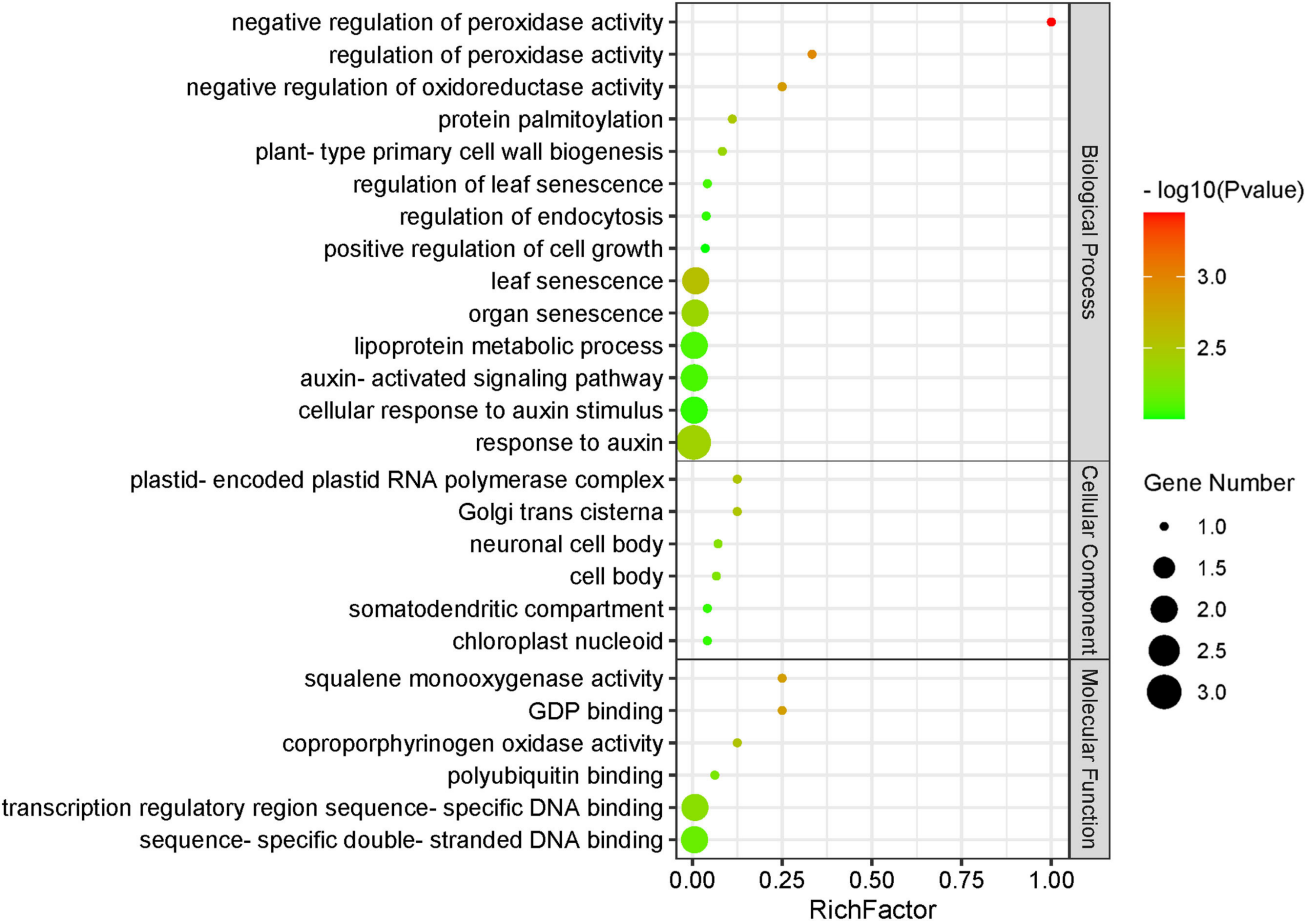
Functional enrichment analysis of protein–protein interaction networks encoded by 14 candidate genes. *P* < 0.01.

Based on the STRING (v11.0) database, we analyzed the interaction network information of 15 key candidate genes. Of these, 14 were obtained using the regulated network, which interacted with various functional proteins by forming a group alone and participated in regulating a variety of biological networks (Figure 4, Supplementary Table S6). For instance, *GRMZM2G149647* (M1), which was annotated as *HSP26* and related to the response to light intensity, was reported with improved chloroplast performance under heat stress by interacting with specific chloroplast proteins (Hu et al., 2015). Meanwhile, the protein HSP101 could promote flowering under normal long-day conditions (Qin et al., 2021). Another gene *GRMZM2G169820* (M9), which was annotated as *ARF21* and interacted with some AUX/IAA-related genes (*AUX27, IAA10*, and *IAA24*), was associated with the root node and root length in maize (Wang, 2016). Furthermore, the candidate gene *GRMZM2G113418* (M13) enhanced drought tolerance and grain yield in field-grown maize and regulated maize inflorescence meristem development *via* redox control of TGA transcriptional activity (Mamoru et al., 2003; Lupini et al., 2016; Tamang et al., 2021; Yang et al., 2021); also, it interacted with *NRT1* and *NRT2.2*, responding to nitrate provision and transport in maize and *Arabidopsis* (Figure 5, Supplementary Table S7).

The aforementioned results showed that the kernel-related traits were affected by *HSPs*, AUX/IAA-related genes, nitrogen transport and provision, and many complex regulations.

## Discussion

### Population Effects in GWAS

With the biotechnology development, GWAS has become an important tool to analyze quantitative traits with big populations and high-throughput molecular markers and provides important candidate genes, including drought stress, primary metabolism, alternative splice, and kernel dehydration in maize (Wen et al., 2016; Zhang et al., 2016; Chen et al., 2018; Li W. et al., 2021). However, the detected loci are limited to the characterization of population, density and frequency of markers, and the computing model and platform, resulting in different conclusions. In this study, AM122 and AM180 were collected and planted in multiple locations in 2019 and 2020 for dissecting the kernel-related size. Using GWAS, we identified 139 unique significant loci, including 36 for KL, 38 for KW, 39 for KT, and 34 for HKW. Among these, only two SNPs (Affx-291390845 and Affx-158854316) were detected in both AM122 and AM180 at the same time, and six SNPs (Affx-291429651, Affx-291384181, Affx-291375641, Affx-159049531, Affx-291397929, and Affx-291399844) were identified using two models. It showed that population differences affected the effectiveness of detection sites despite nearly 60 common inbred lines in AM122 and AM180.

### Candidate Genes Identified Related to Kernel-Related Traits

Some previous studies verified that the genes nearest to, or containing, the associated SNPs were the functional genes, such as *ZmVTE4, ZmVPP1*, and *ZmTIP1* (Li et al., 2012; Ding et al., 2015; Wang et al., 2016; Zhang et al., 2019). Therefore, we defined the genes nearest to the significant associated SNPs as candidate genes. After combining transcriptome data in the B73 reference genome and two maize-inbred lines with different levels of kernel morphology and weight, 15 key genes, which were expressed in kernel development and performed differential expression in the KB182 (small kernel with low weight) and KB020 (big kernel with high weight), were left for the prediction of regulation network and functional annotation. Among these, the genes were annotated as *HSP26* belonging to the putative ubiquitin-conjugating enzyme family, *ARF21*, and glutaredoxin 2, which were involved in peroxidase activity, senescence, and the auxin-related biological process, respectively (Figure 5).

The candidate gene *GRMZM2G149647* (*HSP26*) improved chloroplast performance by responding to light intensity (Hu et al., 2015). *GRMZM2G024693* was associated with KL, which encoded a Ras-related protein and affected the morphology of the Golgi apparatus, thus affecting development by regulating the final processing and packaging of cell secretions (Megumi and Mitsunori, 2015). *GRMZM2G156517* was associated with KL and annotated as a putative ubiquitin assembly enzyme family, which showed high expression during the whole seed development in B73 (Jakobs et al., 2007) (Figure 3B). *GRMZM2G169820* (*ARF21*) associated with KW belonged to auxin response factors, which were occupied by repressive ARF/IAA complexes and regulated a variety of developmental and environmental responses mainly through transcriptional regulation (Chen et al., 2014). *GRMZM2G359521* associated with HKW was mainly involved in regulating organic matter biosynthesis and the metabolic process of proteins, lipids, and small-molecule compounds, which affected grain development. The expression of this gene in the early stage after pollination was higher than that in the late stage (Figure 3). In sum, most of the candidate genes we screened had direct or indirect effects on kernel development and thus affected kernel-related traits. However, the functions of some genes have both not been verified in detail. The mechanism of regulating kernel development in maize needs further exploration. The candidate gene information provided in this study might help in gene cloning related to improving maize yield.

### Application of the Significant Loci

In this study, we found several associated loci located in the 1.5-Mb region on Chromosome 4 (chr4:224307693-225885794), which affected HKW with more than 10% PVE (Supplementary Table S2). This suggested that there were more than one major genes needed investigation in this interval due to the fast decay of linkage disequilibrium in maize. Meanwhile, many studies about the genetic analysis of kernel-related traits were published, and a large number of associated loci were identified. However, few of them were applied in hybrids (Li et al., 2019; Huang et al., 2020; Liu M. et al., 2020). With the help of genotype data of hybrid populations and phenotypic data of kernel size (Li et al., 2022), we found significant differences in kernel size among inbred lines of different genotypes of these associated loci, including Affx-291402221, Affx-291399559, and Affx-291407617 (Supplementary Figure S5). Especially, the genotype AA of Affx-291407617 had a significantly larger KW than the genotypes GG and AG. This illustrated that we could obtain big kernel size while selecting inbred lines by retaining the genotype AA of this SNP. These loci can be used to explore molecular markers for germplasm improvement.

## Conclusions

In this study, four kernel-related traits were investigated in two association panels in 2 years, and GWAS was conducted with two models. A total of 139 significant SNPs associated with kernel-related traits were detected, and 134 candidate genes were located. Combined with the transcriptome data of maize-inbred line B73, 59 candidate genes were found to be expressed in maize seeds after pollination (FPKM ≥ 1). In the meantime, 17 genes with different genotypes, which were differentially expressed in the grains of inbred lines KB182 and KB020, were identified. Combined with the results of the two sets of data, we identified 15 candidate genes. These genes and encoded proteins were found to play an important role in regulating kernel development and storage of material. This study provided important reference information for the exploration of the maize kernel development mechanism and the application of molecular marker–assisted selection (MAS) in high-yield breeding.

## Data Availability Statement

The datasets presented in this study can be found in online repositories. The names of the repository/repositories and accession number(s) can be found in the article/Supplementary Material.

## Author Contributions

SX and JX conceived the research and designed the experiments. ZQ, YW, DH, HL, and FY performed the field experiments and phenotype collection. ZQ performed data analysis and wrote the manuscript. SX and TL modified the manuscript. All authors read and approved the manuscript.

## Funding

This study was supported by the Innovation Project of the National Key R&D Program of China (2018YFD0100200), the National Corn Industry Technology system (CARS-02-77), and the Seed Industry Innovation of Yangling (Ylzy-ym-01).

## Conflict of Interest

The authors declare that the research was conducted in the absence of any commercial or financial relationships that could be construed as a potential conflict of interest.

## Publisher's Note

All claims expressed in this article are solely those of the authors and do not necessarily represent those of their affiliated organizations, or those of the publisher, the editors and the reviewers. Any product that may be evaluated in this article, or claim that may be made by its manufacturer, is not guaranteed or endorsed by the publisher.
