# Supplementary material for: Genome-Wide Association Analysis for Candidate Genes Contributing to Kernel-Related Traits in Maize
Source: Front Plant Sci. 2022 May 24;13:872292. doi: 10.3389/fpls.2022.872292 (PMC9171146; doi:10.3389/fpls.2022.872292)
Supplement: Supplementary file 1 [file Table_1.docx]

**Figure Legend**

**Fig. S1 Correlation between different traits and different locations.**

a: Correlation between different traits and different locations in 2019. b: Correlation between different traits and different locations in 2020. KL: kernel length, KW: kernel width, KT: kernel thickness, HKW: hundred-kernel weight. YL: Yulin, WLMQ: Wulumuqi, NX: Ningxia, ZY: Zhangye, TY: Taiyuan, BLUP : Best Linear Unbiased Prediction. ** indicates significant at the level of P < 0.01, * indicates significant at the level of P < 0.05.

**Fig. S2 Principal component analysis (PCA) of 205 inbred lines (AM205).**

G1-G8: Eight different subpopulations. Different colors represent different subgroups, and arrows represent the position of maize inbred lines KB182 (small kernel) and KB020 (big kernel) in the population structure respectively.

**Fig. S3 Circular Manhattan map of four kernel related traits in 2019.**

a: Circular Manhattan map of corn kernel length(KL) by Farm CPU; b: Circular Manhattan map of corn kernel width(KW) by Farm CPU; c: Circular Manhattan map of corn kernel thickness(KT) by Farm CPU; d: Circular Manhattan map of corn hundred-kernel weight (HKW) by Farm CPU. e: Circular Manhattan map of corn kernel length(KL) by MLM; f: Circular Manhattan map of corn kernel width(KW) by MLM; g: Circular Manhattan map of corn kernel thickness(KT) by MLM; h: Circular Manhattan map of corn hundred-kernel weight (HKW) by MLM. From the inside to the outside, the environment of circular Manhattan map a-d is 19BLUP, 19NX, 19UR, 19YL. Set threshold P = 1×10^-4^.

**Fig. S4 Circular Manhattan map of four kernel related traits in 2020.**

a: Circular Manhattan map of corn kernel length(KL) by Farm CPU; b: Circular Manhattan map of corn kernel width(KW) by Farm CPU; c: Circular Manhattan map of corn kernel thickness(KT) by Farm CPU; d: Circular Manhattan map of corn hundred-kernel weight (HKW) by Farm CPU. e: Circular Manhattan map of corn kernel length(KL) by MLM; f: Circular Manhattan map of corn kernel width(KW) by MLM; g: Circular Manhattan map of corn kernel thickness(KT) by MLM; h: Circular Manhattan map of corn hundred-kernel weight (HKW) by MLM. From the inside to the outside, the environment of circular Manhattan map e-h is 20BLUP, 20NX, 20TY, 20UR, 20ZY. Set threshold P = 1×10^-4^.

**Fig. S5 Analysis of phenotypic differences of different alleles at association sites.**

***indicates significant at the level of P < 0.001, ** indicates significant at the level of P < 0.01, * indicates significant at the level of P < 0.05, ns means not significant.

**Table S1 Summary of phenotype for kernel related traits at seven environments and BLUP.**

**Table S2 Number of all significant loci identified in the study.**

**Table S3 List of candidate genes at the GWAS loci for kernel size related traits in maize.**

**Table S4 Expression of candidate genes during kernel development of maize inbred lines B73.**

S: days after pollination.

**Table S5 Expression of candidate genes during kernel development of maize inbred lines KB182 and KB020.**

DAP: days after pollination.

**Table S6 14 candidate genes encode protein interactions.**

**Table S7 Go enrichment analysis results of 14 candidate genes.**
