# Supplementary figures and images for: Genome-Wide Association Analysis for Candidate Genes Contributing to Kernel-Related Traits in Maize
Source: Front Plant Sci. 2022 May 24;13:872292. doi: 10.3389/fpls.2022.872292 (PMC9171146; doi:10.3389/fpls.2022.872292)

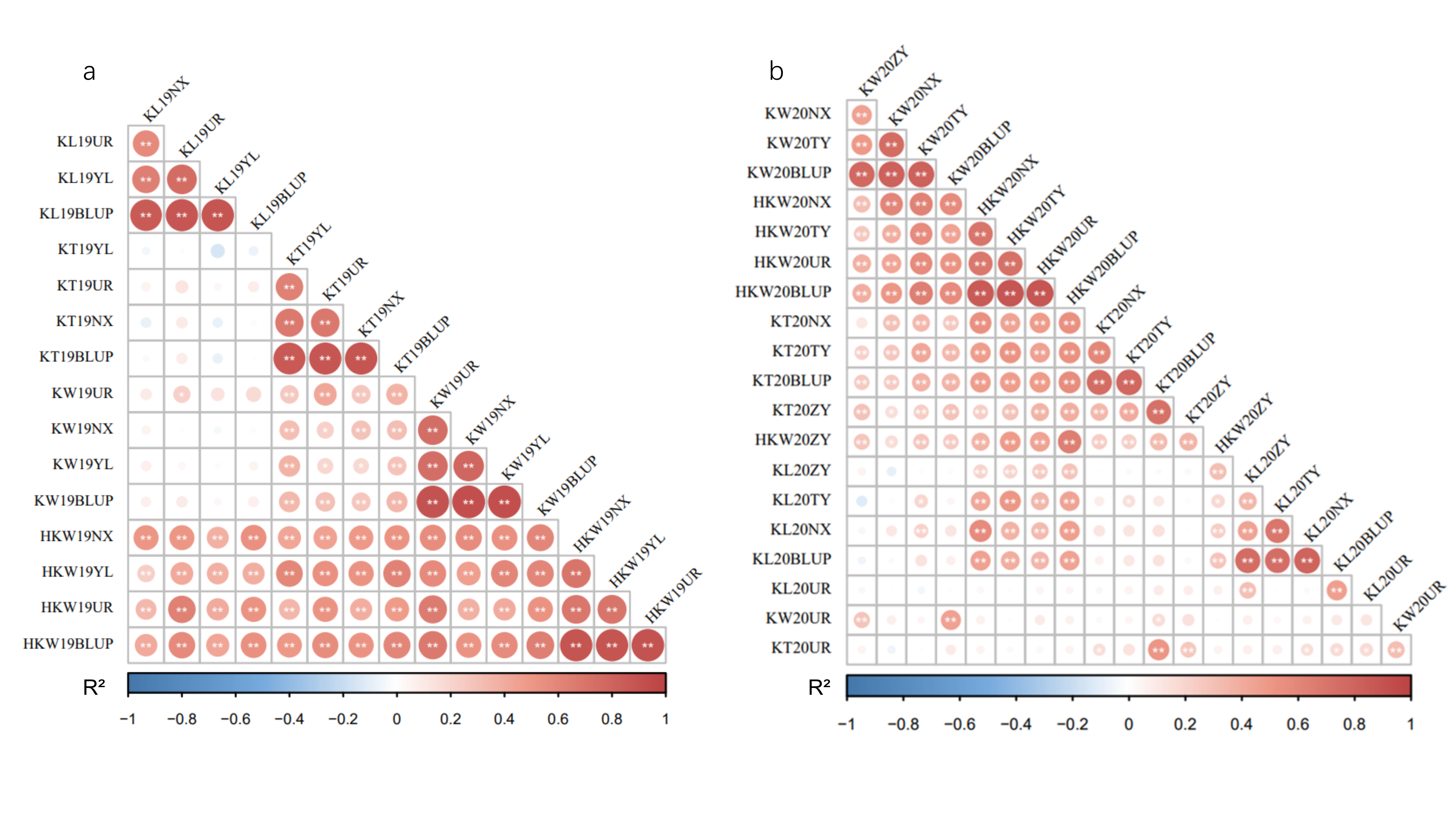

Supplement: Supplementary file 9 [file Image_1.TIFF]

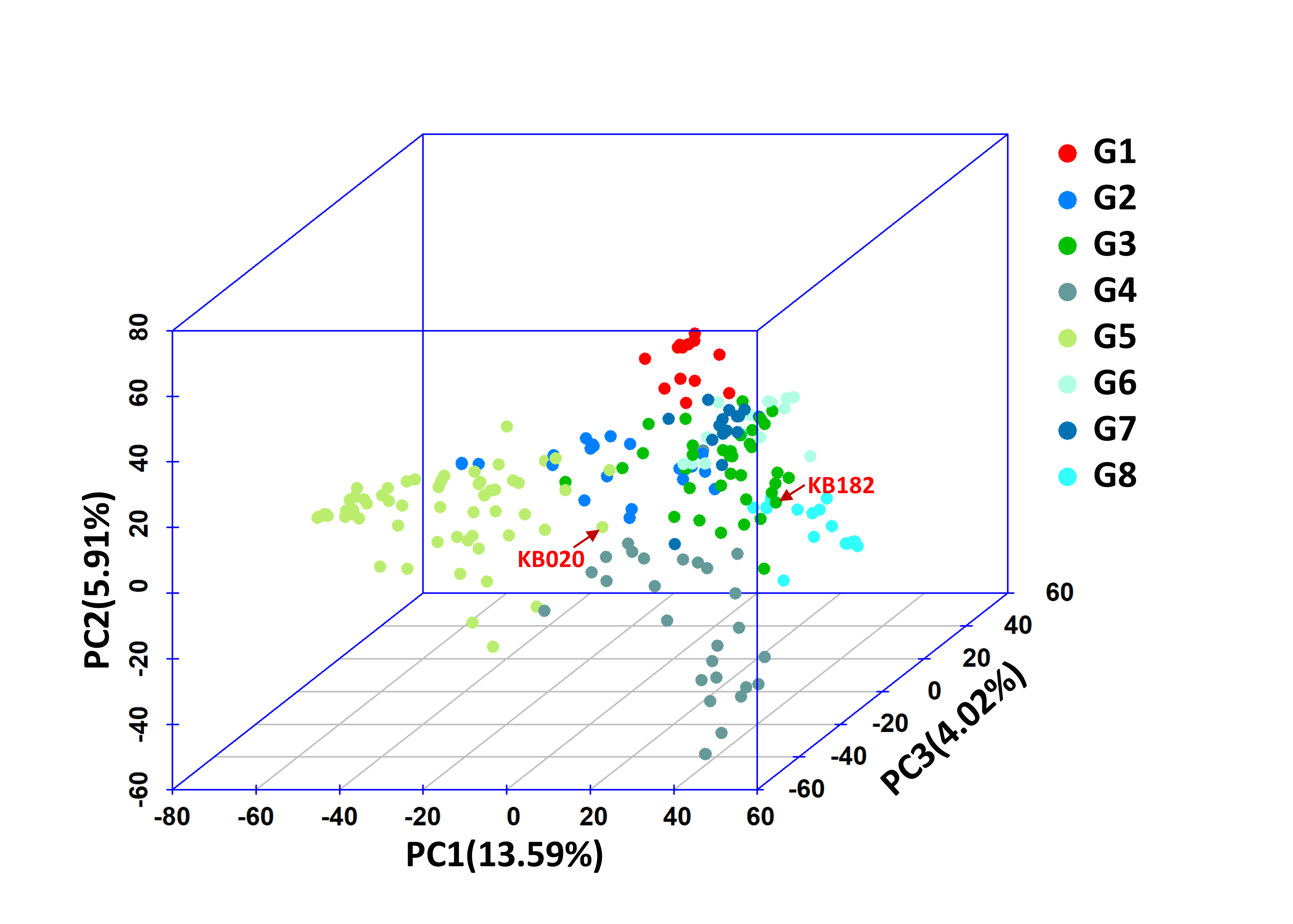

Supplement: Supplementary file 10 [file Image_2.TIFF]

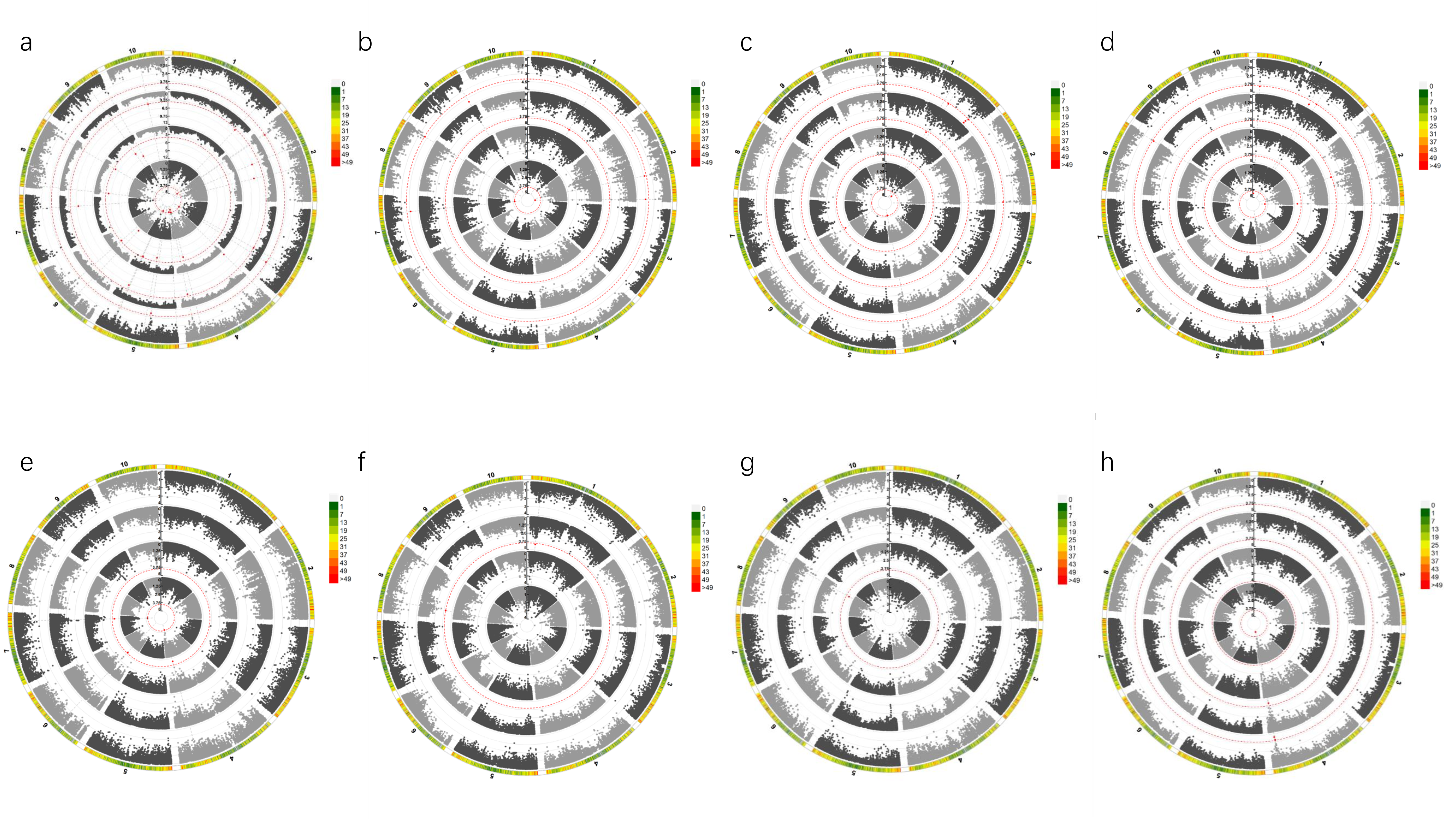

Supplement: Supplementary file 11 [file Image_3.TIFF]

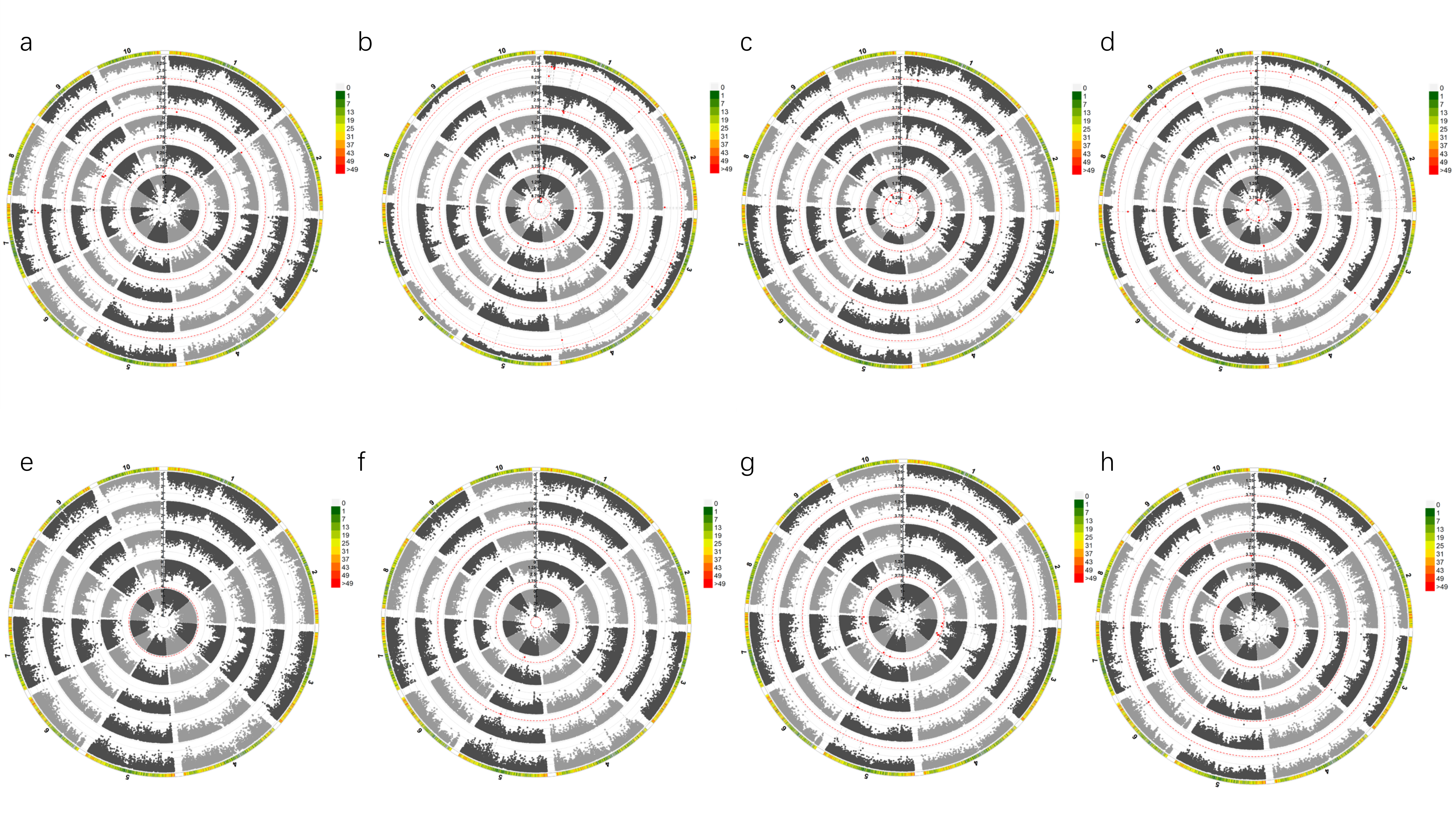

Supplement: Supplementary file 12 [file Image_4.TIFF]

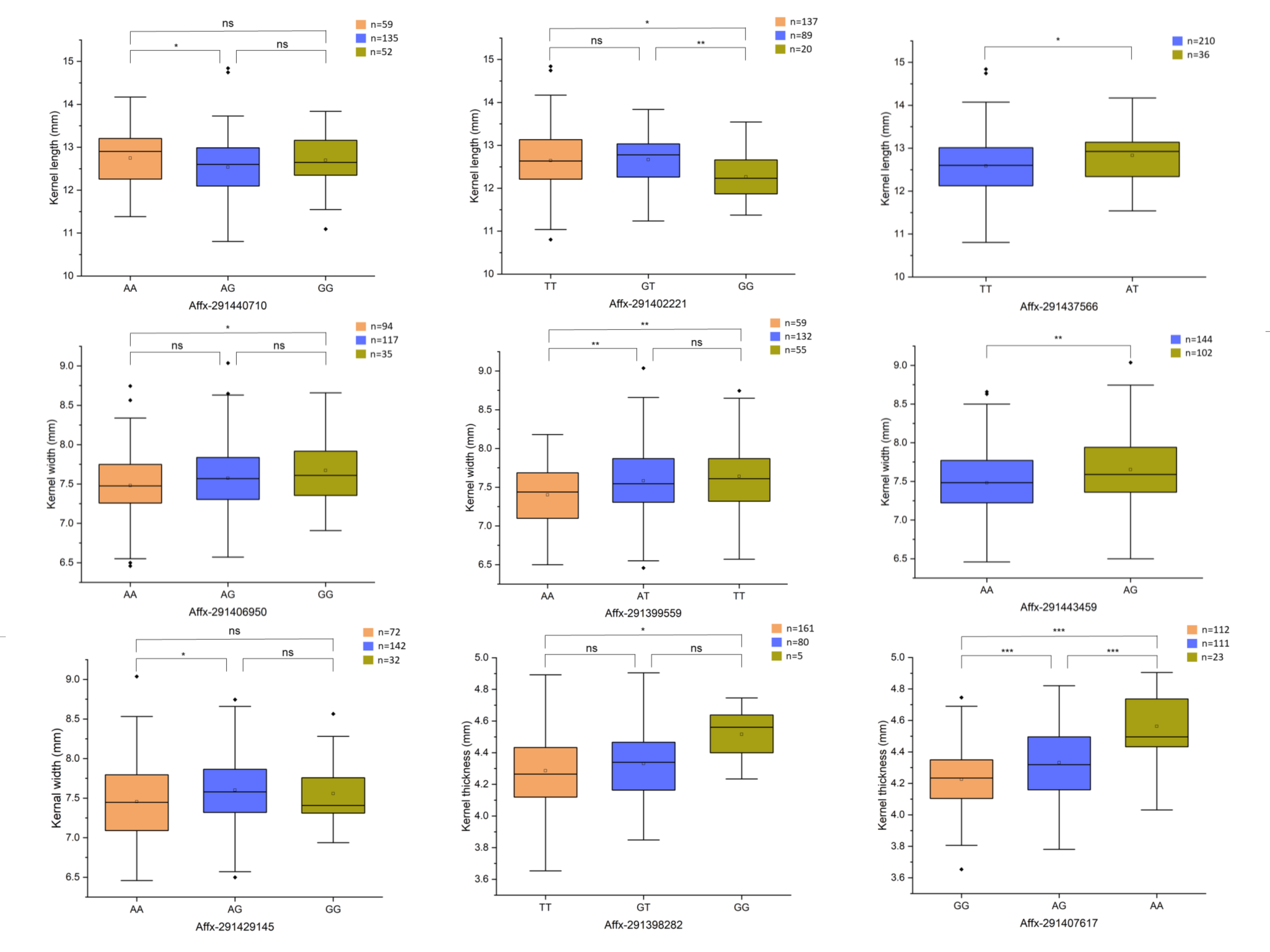

Supplement: Supplementary file 13 [file Image_5.TIFF]
